# Synthesis, crystal structure and absolute configuration of (3a*S*,4*R*,5*S*,7a*R*)-7-(but-3-en-1-yn-1-yl)-2,2-dimethyl-3a,4,5,7a-tetra­hydro-2*H*-1,3-benzodioxole-4,5-diol

**DOI:** 10.1107/S2056989024009733

**Published:** 2024-10-11

**Authors:** Alejandro Peixoto de Abreu Lima, Enrique Pandolfi, Valeria Schapiro, Leopoldo Suescun

**Affiliations:** a Laboratorio de Síntesis Orgánica Departamento de Química Orgánica Facultad de Química Universida de la República Avenida General Flores 2124 CP 11800 Montevideo Uruguay; b Cryssmat-Lab, Cátedra de Física, DETEMA, Facultad de Química, Universidad de la República, Av. General Flores 2124, CP 11800, Montevideo, Uruguay; Universidade Federal do ABC, Brazil

**Keywords:** speciosins, epoxide opening, chiral bicycle, crystal structure

## Abstract

The absolute configuration of the title compound, C_13_H_16_O_4_, determined as 1*S*,2*R*,3*S*,4*R* based on the synthetic pathway, was confirmed by single-crystal X-ray diffraction. The mol­ecule is a relevant inter­mediary for the synthesis of speciosins, ep­oxy­quinoides or their analogues. The mol­ecule contains fused five- and six-membered rings with two free hydroxyl groups and two protected as an iso­propyl­idenedioxo ring. The packing is directed by hydrogen bonds that define double planes of mol­ecules laying along the *ab* plane and van der Waals inter­actions between aliphatic chains that point outwards of the planes.

## Abstract 2

The crystal structure of the title compound was solved to confirm the relative absolute configuration of the chiral centers.

## 1. Chemical context

**Figure 1:**
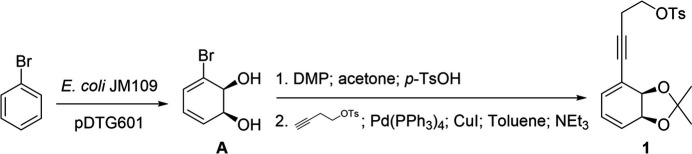
Biotransformation of bromo­benzene and alkyne side chain introduction *via* Sonogashira coupling.

**Figure 2:**
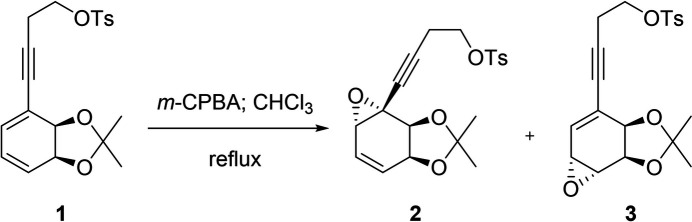
Epoxidation of mol­ecule **1**.

**Figure 3:**
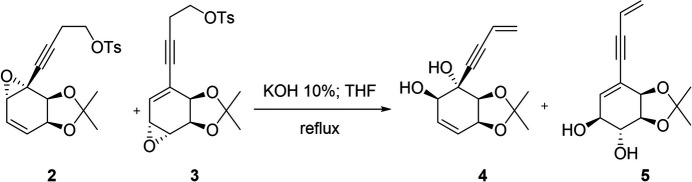
Epoxide opening in basic aqueous conditions.

Speciosins are a group of ep­oxy­quinoids isolated from fungal origins that exhibit diverse biological activities (Jiang *et al.*, 2009 ▸, 2011 ▸; Kim *et al.*, 2006 ▸). So far, only one racemic synthesis has been reported (Hookins *et al.*, 2011 ▸). We proposed the first synthetic enanti­oselective approach towards speciosins starting from halodiols obtained from halo­benzene (Vila *et al.*, 2013 ▸). In our reported enanti­oselective route for the synthesis of speciosin A, we obtained mol­ecule **1** in two steps in 65% yield from the diol *A* (Fig. 1 ▸). Compound **1** had to be epoxidized in order to functionalize the most substituted double bond (Peixoto de Abreu Lima *et al.*, 2019 ▸). That reaction yielded epoxides **2** and **3**, a pair of regioisomers. Despite our efforts, we could not obtain the desired regioselectivity towards **2**, and we obtained a mixture of **2** and **3** using a wide variety of solvents, temperature, and concentration (Fig. 2 ▸). Another problem we faced was the separation of **2** and **3**, which obliged us to continue our synthetic route using the mixture of both epoxides. Treating **2** and **3** under basic conditions we obtained the products of tosyl­ate elimination and epoxide basic opening for each regioisomer (Fig. 3 ▸). Diols **4** and **5** could be successfully separated by column chromatography. Compound **4** is a valuable inter­mediate for the synthesis of speciosin A. On the other hand, **5** may be useful as an inter­mediate for the synthesis of other ep­oxy­quinoides like harveynone or its analogues (Pandolfi *et al.*, 2013 ▸; Nagata *et al.*, 1992 ▸), especially given that it could easily be obtained in higher proportions (Peixoto de Abreu Lima *et al.*, 2019 ▸). The epoxide opening in basic aqueous conditions can yield two diastereomers depending on which carbon is attacked by the hydroxyl, though it is expected that the allylic position is more reactive. Thus, it is important to confirm the stereochemistry of the formed diol before continuing with further synthetic steps by means of crystallization and crystal structure determination.
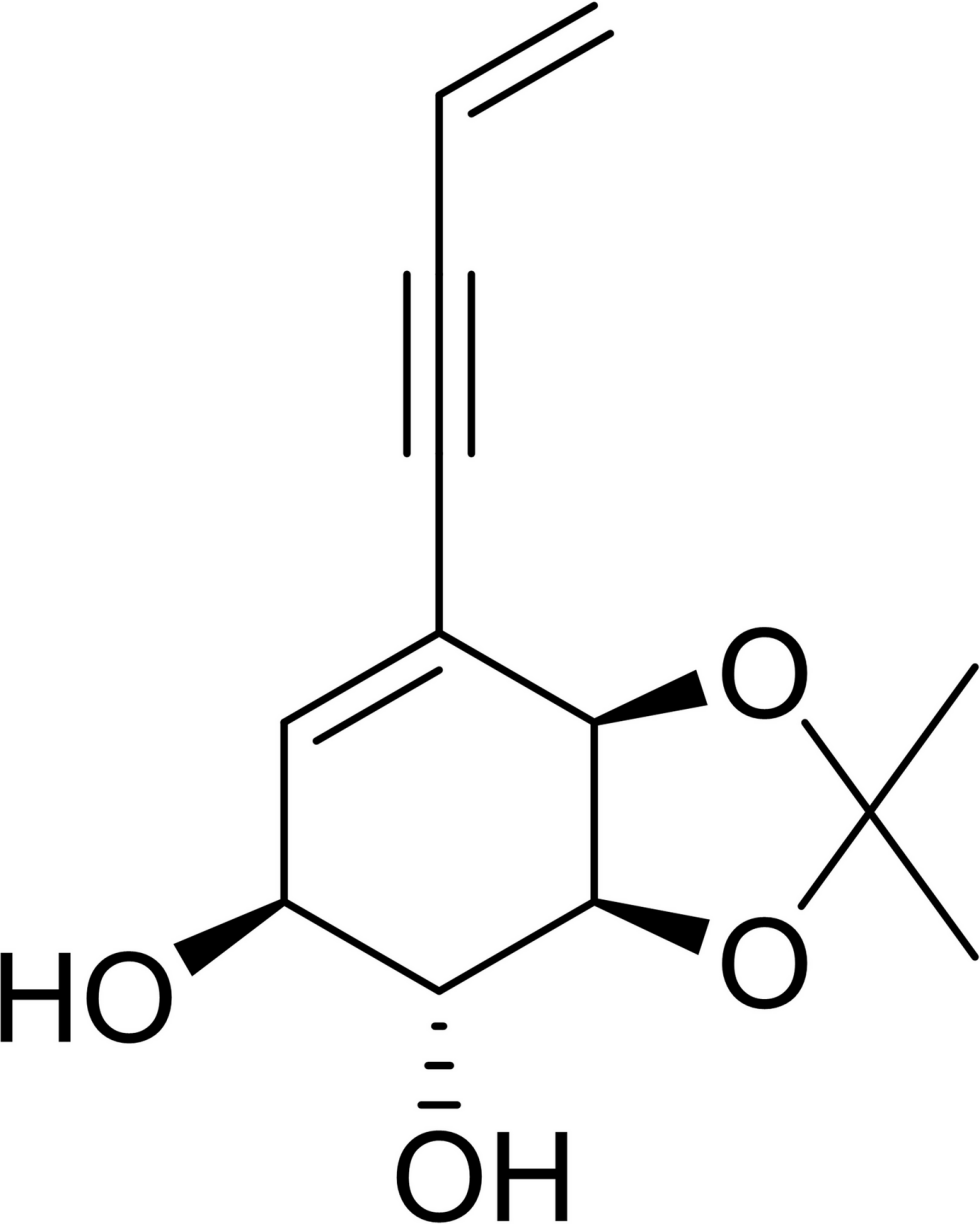


## 2. Structural commentary

**Figure 4:**
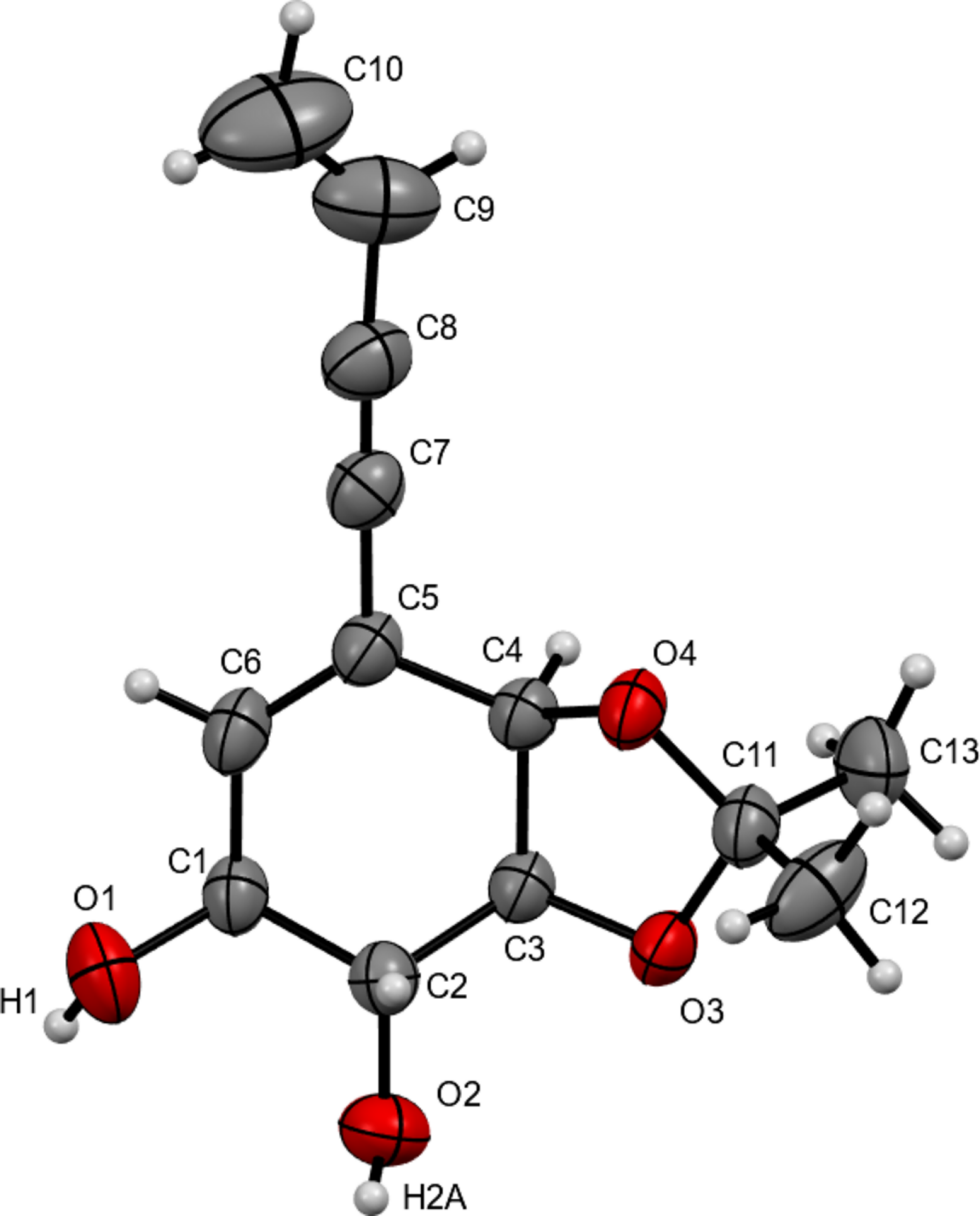
*ORTEP* view of the title compound showing the numbering scheme. Ellipsoids are drawn at the 30% probability level.

Fig. 4 ▸ shows the structure of **5** (the title compound) as determined by single-crystal X-ray diffraction. All bond distances and angles fall within the expected values for this kind of organic mol­ecule. The absolute configuration, determined as 1*S*,2*R*,3*S*,4*R* based on the synthetic pathway, was confirmed by X-ray diffraction on the basis of anomalous dispersion of light atoms only. The cyclo­hexene core of the mol­ecule shows puckering angles (Cremer & Pople, 1975 ▸) of Θ = 61.0 (7)° and Φ = 51.7 (8)°, indicating a conformation between envelope (Θ = 54.7°, Φ = 60°) and screw-boat (Θ = 67.5°, Φ = 30°) with C2 forming the flap. The planar part of the C1–C6–C5–C4–C3 ring (fit of the plane with an r.m.s. deviation of 0.0405 Å) forms a dihedral angle of 52.5 (3)° with the flap (C1–C2–C3). The linear buten-3-en-1-ynyl chain is coplanar with the main part of the ring with a maximum deviation from the plane of 0.21 Å for C10 (see supporting information). The five-membered iso­propyl­idenedioxo ring (O3–C3–C4–O4–C11) is also envelope-shaped with Φ_2_ = 289.1 (10)° (idealized Φ_2_ = 288°) with O4 forming the flap. The absolute configuration of the four chiral centers and the double bond allows for the five substituents of the cyclo­hexene ring to be equatorial (O1, O2, C7) or bis­ectional (O3, O4) with three of the five H atoms being in axial positions.

## 3. Supra­molecular features

**Figure 5:**
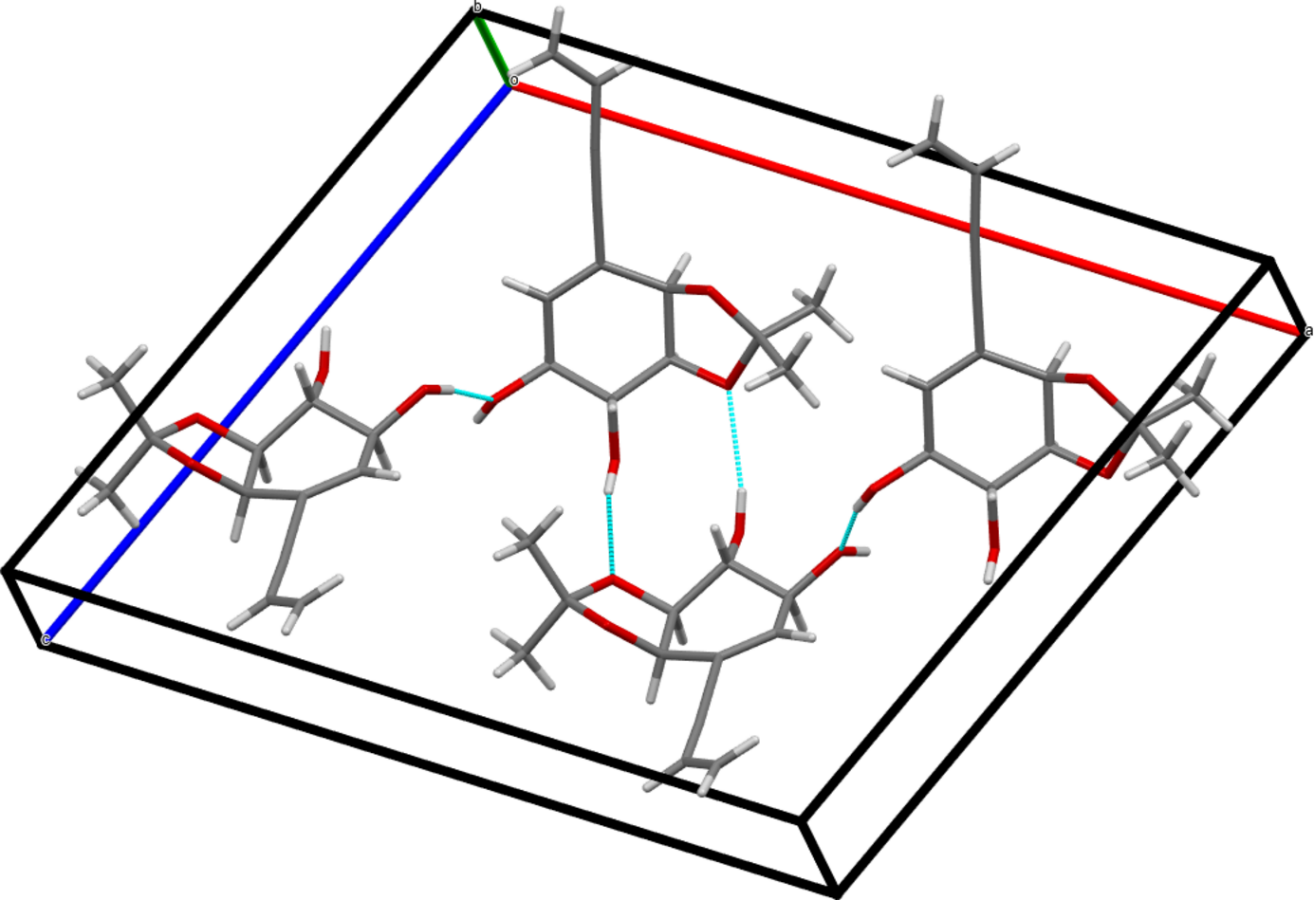
Unit-cell contents of **5**. Note that two hydrogen bonds involving O2 and O3 connect twofold-rotation-related mol­ecules, and a third one connects screw-rotation-related mol­ecules.

**Figure 6:**
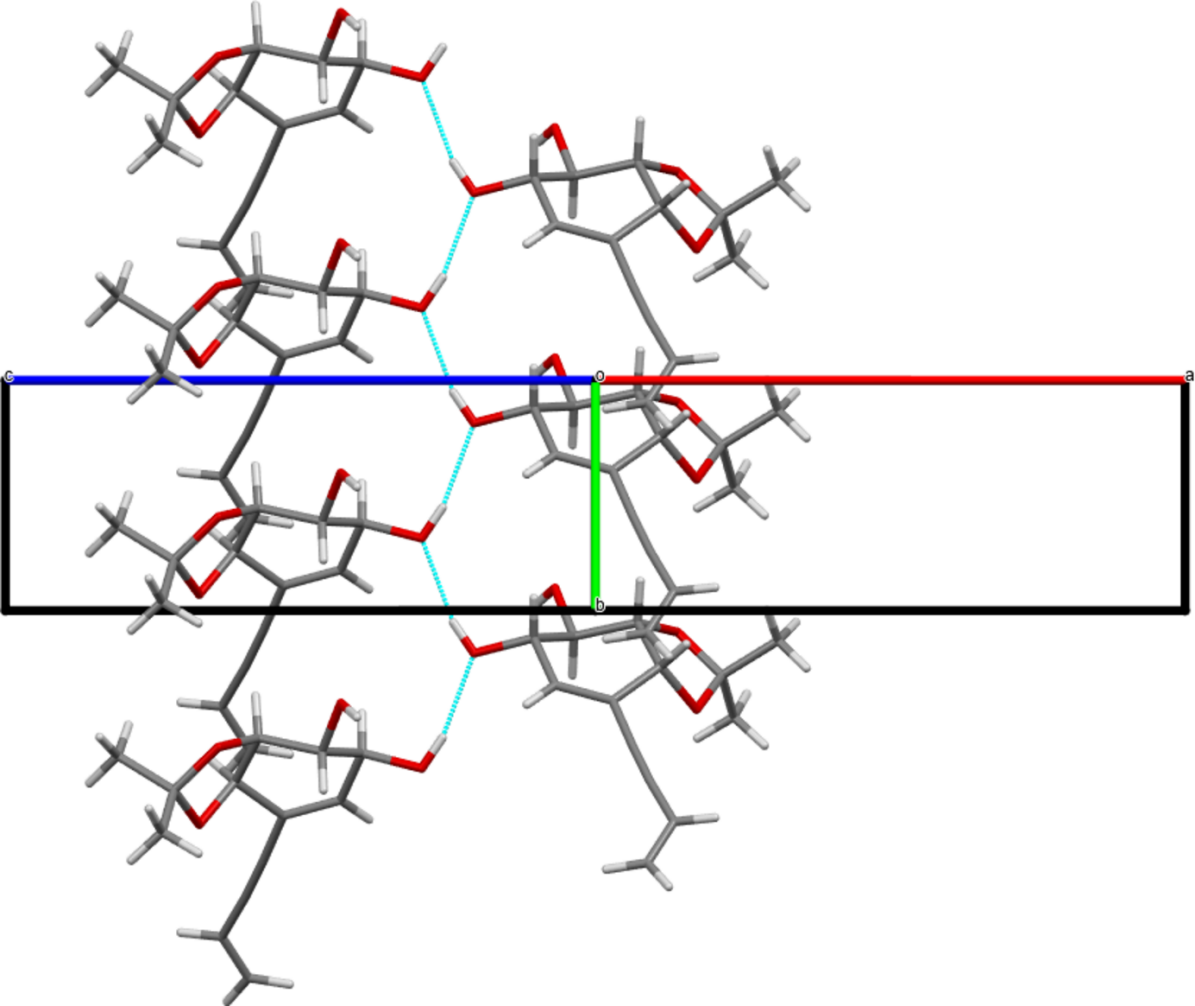
View of the unit cell along the [101] direction showing linear chains of mol­ecules connected through O1—H1⋯O1 hydrogen bonds.

**Figure 7:**
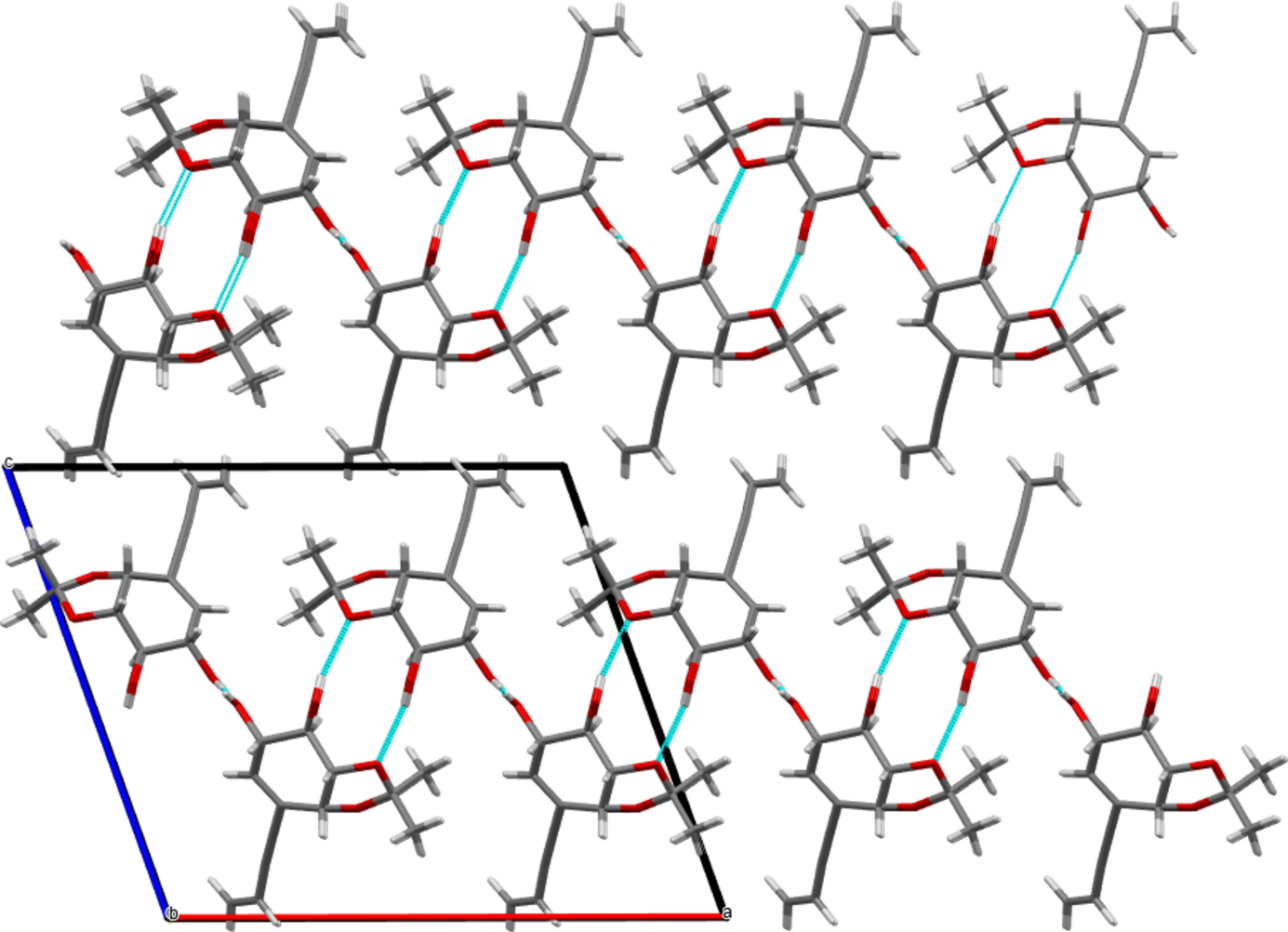
View of the unit cell along [010] direction showing the weak inter­actions between aliphatic chains in parallel hydrogen-bonded mol­ecular planes of **5**.

In the crystal, mol­ecules are connected through strong O—H⋯O hydrogen bonds (see Table 1 ▸ and Fig. 5 ▸). Pairs of O2—H2*A*⋯O3^i^ hydrogen bonds connect mol­ecules related by a twofold rotation along [010]. O1—H1 groups pointing outwards from the axis form infinite O1—H1⋯O1^ii^ chains along [010] between twofold-screw-rotated equivalent mol­ecules [symmetry codes: (i) 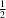 − *x*, *y* − 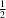, 1 − *z*; (ii) 1 − *x*, *y*, 1 − *z*] as shown in Fig. 6 ▸. The combination of both hydrogen bonds provides a strong set of inter­molecular inter­actions that define planes parallel to the *ac* plane with the but-3-en-1-ynyl chains pointing outwards on both sides. Weak van der Waals inter­actions connect these planes along [001] as shown in Fig. 7 ▸.

## 4. Database survey

One dozen entries in the 2024.1 version of the CSD (Groom *et al.*, 2016 ▸) are related to the title compound sharing a mono-substituted tetra­hydroxy-cyclo­hexene core. The most inter­esting ones are discussed hereafter. The asymmetric unit of TEDZUA (Buckler *et al.*, 2017 ▸) contains two independent mol­ecules that only differ from **5** in the substituent at C5 sharing the same absolute structure at all the chiral centers and the iso­propyl­idenedioxo ring containing O3 and O4. The cyclo­hexene cores show an almost perfect envelope conformation in both mol­ecules, but the shape of the bulky vanillin substituent affects the conformation of the five-membered ring and the positions of O4 substituents of C4 in each mol­ecule making them axial. The packing is directed by π-stacking between vanillin residues and a more complex hydrogen-bond network including one crystallization water mol­ecule. RAQLED (Taher *et al.*, 2017 ▸) is a tetra­hydroxy-cyclo­hexene with a –C*sp*–C*sp*– chain at C5 (ending in a substituted benzene ring) but the hydroxyl groups at C3 and C4 are free (not part of a five-membered ring) and C2 has an inverted absolute configuration making the cyclo­hexene ring fall between an envelope and a half-chair conformation with two of the OH substituents on C4 and C1 in axial positions. The packing is similar to that of **5** with a complex hydrogen-bond network defining planes of mol­ecules with the non-polar substituents pointing outwards, inter­acting weakly with parallel planes. RURVEH (Macías *et al.*, 2015 ▸) and HOYFIN (Tibhe *et al.*, 2018 ▸) show a tetra­hydroxy-cyclo­hexene core with inverted configurations at C1 and C2 with respect to **5**. The ring conformations fall between envelope and half-chair with the flap at the opposite side of the ring plane, keeping both OH substituents close to equatorial positions. RURVEH also shares the iso­propyl­idenedioxo ring including O3 and O4 with **5**. The packing in HOYFIN is directed by a complex 3D hydrogen-bond network while two different inter­molecular hydrogen bonds define the packing of RURVEH, but in this case linear chains of hydrogen-bonded mol­ecules are observed with weak inter­actions between parallel chains.

## 5. Synthesis and crystallization

The synthesis of the title compound was carried out through a mixture of epoxides **2** and **3**. The epoxides (400 mg; 1.00 mmol, ratio **2**:**3** 5:1) were dissolved in tetra­hydro­furan (40 mL) at room temperature and a solution of potassium hydroxide 10% mV (40 mL) was added (Fig. 3 ▸). The mixture was refluxed for 4 h. After completion of the reaction, the mixture was diluted with 50 mL of ethyl acetate. The aqueous fraction was neutralized with 10% HCl and extracted with ethyl acetate (portions of 20 mL) until no further products were seen on the aqueous phase on TLC. The combined ethereal fractions were dried with Na_2_SO_4_ and filtered. Concentration of the filtrate, followed by column chromatography (hexa­nes:ethyl acetate 7:3) yielded **4** (177 mg; 81%) and **5** (33 mg; 15%). Small, plate-shaped crystals suitable for X-ray structure analysis were obtained by dissolving **5** in MeOH and slowly evaporating the solvent at room temperature, (m.p.) dec. 435 K. HRMS: C_13_H_16_O_4_+Na calc:259.0946; exp : 259.0953. [α]^25.5^_589nm_ = −13.10° (0.32 g/100 mL of MeOH). ^1^H NMR (400 MHz, CDCl_3_) δ (ppm): 6.18 (*d*, *J* = 2.1 Hz, 1H), 5.94 (*dd*, *J* = 17.6, 11.2 Hz, 1H), 5.71 (*dd*, *J* = 17.6, 2.0 Hz, 1H), 5.54 (*dd*, *J* = 11.2, 2.1 Hz, 1H), 4.61 (*d*, *J* = 6.4 Hz, 1H), 4.18 (*d*, *J* = 8.5 Hz, 1H), 4.12 (*dd*, *J* = 8.7, 6.3 Hz, 1H), 3.66 (*t*, *J* = 8.5 Hz, 1H), 2.90 (*br s*, 2H), 1.54 (*s*, 3H), 1.43 (*s*, 3H). ^13^C NMR (101 MHz, CDCl_3_) δ (ppm): 138.6, 128.0, 119.2, 117.0, 111.1, 89.3, 87.3, 77.4, 74.6, 74.2, 70.4, 28.3, 26.1.

## 6. Refinement

Crystal data, data collection and structure refinement details are summarized in Table 2 ▸. All C and O atoms in the structure were refined anisotropically with restraints applied to the thermal ellipsoids of C9 and C10 and their bond distance that appeared shorter than expected due to significant librational disorder of the final atoms of the chain. Neither C9 nor C10 were split due to the lack of clear alternative positions. All H atoms were located in difference Δ*F* maps and refined freely in the initial model to confirm the absolute structure of the chiral centers. In the final model they were modeled in geometrically suitable positions and refined as riding with *U*_iso_=1.2/1.5*U*_eq_ of the core/terminal parent atom.

## Supplementary Material

Crystal structure: contains datablock(s) I. DOI: 10.1107/S2056989024009733/ee2008sup1.cif

Structure factors: contains datablock(s) I. DOI: 10.1107/S2056989024009733/ee2008Isup2.hkl

Supporting information file. DOI: 10.1107/S2056989024009733/ee2008Isup3.cml

CCDC reference: 2389407

Additional supporting information:  crystallographic information; 3D view; checkCIF report

## Figures and Tables

**Table 1:** Hydrogen-bond geometry (Å, °)

*D*—H⋯*A*	*D*—H	H⋯*A*	*D*⋯*A*	*D*—H⋯*A*
O1—H1⋯O1^i^	0.82	2.00	2.803 (3)	167
O2—H2*A*⋯O3^ii^	0.82	2.19	2.995 (6)	168

Symmetry codes: (i) 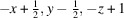; (ii) 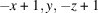.

**Table 2:** Experimental details

Crystal data	
Chemical formula	C_13_H_16_O_4_
*M*_r_	236.26
Crystal system, space group	Monoclinic, *C*2
Temperature (K)	296
*a*, *b*, *c* (Å)	17.4126 (13), 5.0996 (4), 14.9856 (11)
β (°)	109.652 (2)
*V* (Å^3^)	1253.17 (16)
*Z*	4
Radiation type	Cu *K*α
μ (mm^−1^)	0.77
Crystal size (mm)	0.38 × 0.26 × 0.12

Data collection	
Diffractometer	Bruker D8 Venture/Photon 100 CMOS
Absorption correction	Multi-scan (*SADABS*; Krause *et al.*, 2015 ▸)
*T*_min_, *T*_max_	0.646, 0.754
No. of measured, independent and observed [*I* > 2σ(*I*)] reflections	10420, 2628, 2138
*R*_int_	0.049
(sin θ/λ)_max_ (Å^−1^)	0.642

Refinement	
*R*[*F*^2^ > 2σ(*F*^2^)], *wR*(*F*^2^), *S*	0.080, 0.248, 1.13
No. of reflections	2628
No. of parameters	157
No. of restraints	3
H-atom treatment	H-atom parameters constrained
Δρ_max_, Δρ_min_ (e Å^−3^)	0.41, −0.21
Absolute structure	Flack *x* determined using 752 quotients [(*I*^+^)−(*I*^−^)]/[(*I*^+^)+(*I*^−^)] (Parsons *et al.*, 2013 ▸)
Absolute structure parameter	0.1 (4)

Computer programs: *APEX3* and *SAINT* (Bruker, 2018 ▸), *OLEX2.solve* (Bourhis *et al.*, 2015 ▸, Dolomanov *et al.*, 2009 ▸), *SHELXL2019/2* (Sheldrick, 2015 ▸) and *ShelXle* (Hübschle *et al.*, 2011 ▸).

## supplementary crystallographic information

### (3aS,4R,5S,7aR)-7-(But-3-en-1-yn-1-yl)-2,2-dimethyl-3a,4,5,7a-tetrahydro-2H-1,3-benzodioxole-4,5-diol Crystal data

**Table:**

C_13_H_16_O_4_	*F*(000) = 504
*M**_r_* = 236.26	*D*_x_ = 1.252 Mg m^−^^3^
Monoclinic, *C*2	Cu *K*α radiation, λ = 1.54178 Å
*a* = 17.4126 (13) Å	Cell parameters from 7392 reflections
*b* = 5.0996 (4) Å	θ = 5.2–77.8°
*c* = 14.9856 (11) Å	µ = 0.77 mm^−^^1^
β = 109.652 (2)°	*T* = 296 K
*V* = 1253.17 (16) Å^3^	Plate, colourless
*Z* = 4	0.38 × 0.26 × 0.12 mm

### (3aS,4R,5S,7aR)-7-(But-3-en-1-yn-1-yl)-2,2-dimethyl-3a,4,5,7a-tetrahydro-2H-1,3-benzodioxole-4,5-diol Data collection

**Table:**

Bruker D8 Venture/Photon 100 CMOS diffractometer	2628 independent reflections
Radiation source: Cu Incoatec microsource	2138 reflections with *I* > 2σ(*I*)
Detector resolution: 10.4167 pixels mm^-1^	*R*_int_ = 0.049
\j and ω scans	θ_max_ = 81.6°, θ_min_ = 5.3°
Absorption correction: multi-scan (SADABS; Krause *et al.*, 2015)	*h* = −22→22
*T*_min_ = 0.646, *T*_max_ = 0.754	*k* = −6→6
10420 measured reflections	*l* = −18→19

### (3aS,4R,5S,7aR)-7-(But-3-en-1-yn-1-yl)-2,2-dimethyl-3a,4,5,7a-tetrahydro-2H-1,3-benzodioxole-4,5-diol Refinement

**Table:**

Refinement on *F*^2^	Hydrogen site location: inferred from neighbouring sites
Least-squares matrix: full	H-atom parameters constrained
*R*[*F*^2^ > 2σ(*F*^2^)] = 0.080	*w* = 1/[σ^2^(*F*_o_^2^) + (0.1316*P*)^2^ + 0.6357*P*] where *P* = (*F*_o_^2^ + 2*F*_c_^2^)/3
*wR*(*F*^2^) = 0.248	(Δ/σ)_max_ < 0.001
*S* = 1.13	Δρ_max_ = 0.41 e Å^−^^3^
2628 reflections	Δρ_min_ = −0.21 e Å^−^^3^
157 parameters	Absolute structure: Flack *x* determined using 752 quotients [(*I*^+^)-(*I*^-^)]/[(*I*^+^)+(*I*^-^)] (Parsons *et al.*, 2013)
3 restraints	Absolute structure parameter: 0.1 (4)

### (3aS,4R,5S,7aR)-7-(But-3-en-1-yn-1-yl)-2,2-dimethyl-3a,4,5,7a-tetrahydro-2H-1,3-benzodioxole-4,5-diol Special details

**Table:**

Geometry. All esds (except the esd in the dihedral angle between two l.s. planes) are estimated using the full covariance matrix. The cell esds are taken into account individually in the estimation of esds in distances, angles and torsion angles; correlations between esds in cell parameters are only used when they are defined by crystal symmetry. An approximate (isotropic) treatment of cell esds is used for estimating esds involving l.s. planes.Least-squares planes (x,y,z in crystal coordinates) and deviations from them (* indicates atom used to define plane) 7.1271 (0.0500) x + 3.5828 (0.0094) y + 6.1525 (0.0364) z = 4.8815 (0.0081) * -0.0395 (0.0049) C1 * 0.0862 (0.0063) C2 * -0.0383 (0.0049) C4 * -0.0247 (0.0051) C5 * 0.0316 (0.0054) C6 * 0.0183 (0.0075) C7 * -0.0331 (0.0100) C8 * -0.1482 (0.0159) C9 * 0.1478 (0.0145) C10 0.7181 (0.0077) C3 1.0769 (0.0080) O1 0.9312 (0.0104) O2 0.6098 (0.0104) O3 0.5627 (0.0104) O4 Rms deviation of fitted atoms = 0.0798 6.7576 (0.0357) x + 3.6373 (0.0092) y + 6.2811 (0.0338) z = 4.8211 (0.0091) Angle to previous plane (with approximate esd) = 1.364 ( 0.435 ) * -0.0646 (0.0036) C1 * 0.0457 (0.0028) C2 * -0.0264 (0.0029) C4 * 0.0026 (0.0042) C5 * 0.0427 (0.0038) C6 0.6990 (0.0071) C3 0.0448 (0.0094) C7 0.0054 (0.0126) C8 -0.0921 (0.0258) C9 0.2316 (0.0289) C10 1.0394 (0.0069) O1 0.8638 (0.0074) O2 0.5755 (0.0096) O3 0.5959 (0.0094) O4 Rms deviation of fitted atoms = 0.0419 - 1.1053 (0.0796) x - 4.9917 (0.0065) y + 3.0656 (0.0891) z = 0.2849 (0.0528) Angle to previous plane (with approximate esd) = 52.465 ( 0.318 ) * 0.0000 (0.0000) C2 * 0.0000 (0.0000) C3 * 0.0000 (0.0000) C4 Rms deviation of fitted atoms = 0.0000

### (3aS,4R,5S,7aR)-7-(But-3-en-1-yn-1-yl)-2,2-dimethyl-3a,4,5,7a-tetrahydro-2H-1,3-benzodioxole-4,5-diol Fractional atomic coordinates and isotropic or equivalent isotropic displacement parameters (Å^2^)

**Table:**

	*x*	*y*	*z*	*U*_iso_*/*U*_eq_	
C2	0.3746 (3)	0.1116 (12)	0.4110 (3)	0.0683 (11)	
H2	0.398970	0.279483	0.437829	0.082*	
C1	0.2844 (3)	0.1188 (12)	0.3889 (4)	0.0709 (12)	
H1A	0.261212	−0.053188	0.365494	0.085*	
O1	0.2645 (3)	0.1853 (10)	0.4702 (3)	0.0957 (14)	
H1	0.252857	0.051859	0.493396	0.144*	
O3	0.47692 (19)	0.0935 (9)	0.3375 (3)	0.0779 (11)	
O4	0.4120 (2)	0.4298 (8)	0.2405 (3)	0.0750 (10)	
C3	0.3927 (3)	0.0527 (10)	0.3215 (3)	0.0635 (11)	
H3	0.377713	−0.128755	0.301629	0.076*	
C4	0.3504 (3)	0.2420 (11)	0.2399 (3)	0.0661 (11)	
H4	0.333731	0.145670	0.179805	0.079*	
C5	0.2776 (3)	0.3908 (12)	0.2485 (4)	0.0714 (12)	
O2	0.4080 (3)	−0.0979 (11)	0.4766 (3)	0.0939 (13)	
H2A	0.433176	−0.036444	0.528898	0.141*	
C6	0.2469 (3)	0.3234 (12)	0.3150 (5)	0.0791 (15)	
H6	0.199730	0.407119	0.315916	0.095*	
C7	0.2395 (3)	0.5872 (13)	0.1767 (4)	0.0790 (14)	
C8	0.2085 (4)	0.7380 (16)	0.1167 (5)	0.0968 (19)	
C9	0.1732 (7)	0.918 (2)	0.0403 (7)	0.139 (4)	
H9	0.190917	0.900463	−0.011464	0.167*	
C10	0.1213 (9)	1.096 (3)	0.0339 (11)	0.197 (7)	
H10A	0.101128	1.123418	0.083313	0.295*	
H10B	0.103243	1.200024	−0.020116	0.295*	
C11	0.4859 (3)	0.2806 (12)	0.2714 (4)	0.0750 (14)	
C12	0.5552 (4)	0.4617 (17)	0.3208 (8)	0.119 (3)	
H12A	0.562490	0.585508	0.276005	0.179*	
H12B	0.604292	0.361554	0.347503	0.179*	
H12C	0.543123	0.553832	0.370311	0.179*	
C13	0.5005 (5)	0.131 (2)	0.1907 (5)	0.113 (3)	
H13A	0.459372	−0.001682	0.167864	0.170*	
H13B	0.553287	0.049946	0.213143	0.170*	
H13C	0.497907	0.250061	0.140183	0.170*	

### (3aS,4R,5S,7aR)-7-(But-3-en-1-yn-1-yl)-2,2-dimethyl-3a,4,5,7a-tetrahydro-2H-1,3-benzodioxole-4,5-diol Atomic displacement parameters (Å^2^)

**Table:**

	*U*^11^	*U*^22^	*U*^33^	*U*^12^	*U*^13^	*U*^23^
C2	0.064 (2)	0.073 (3)	0.070 (2)	−0.002 (2)	0.024 (2)	0.004 (2)
C1	0.064 (3)	0.072 (3)	0.085 (3)	−0.001 (2)	0.037 (2)	0.002 (3)
O1	0.113 (3)	0.091 (3)	0.109 (3)	−0.006 (3)	0.071 (3)	−0.009 (2)
O3	0.0525 (16)	0.093 (3)	0.090 (2)	0.0072 (17)	0.0260 (15)	0.016 (2)
O4	0.0591 (17)	0.073 (2)	0.100 (2)	0.0048 (16)	0.0359 (16)	0.0134 (19)
C3	0.054 (2)	0.064 (3)	0.074 (3)	0.0032 (19)	0.0240 (19)	0.001 (2)
C4	0.058 (2)	0.069 (3)	0.071 (2)	−0.001 (2)	0.0226 (19)	0.000 (2)
C5	0.057 (2)	0.072 (3)	0.084 (3)	0.005 (2)	0.023 (2)	0.004 (3)
O2	0.102 (3)	0.099 (3)	0.076 (2)	0.009 (3)	0.0231 (19)	0.018 (2)
C6	0.058 (2)	0.077 (3)	0.109 (4)	−0.001 (2)	0.037 (3)	0.003 (3)
C7	0.068 (3)	0.079 (3)	0.087 (3)	−0.005 (3)	0.021 (2)	−0.004 (3)
C8	0.078 (4)	0.098 (4)	0.103 (4)	−0.003 (4)	0.015 (3)	0.010 (4)
C9	0.158 (9)	0.114 (7)	0.116 (6)	−0.019 (6)	0.008 (6)	0.016 (6)
C10	0.196 (13)	0.124 (9)	0.219 (14)	0.009 (9)	0.002 (10)	0.057 (11)
C11	0.063 (3)	0.075 (3)	0.096 (3)	0.002 (2)	0.038 (2)	0.008 (3)
C12	0.069 (3)	0.086 (5)	0.183 (8)	−0.008 (3)	0.016 (4)	0.008 (5)
C13	0.111 (5)	0.138 (6)	0.111 (5)	0.035 (5)	0.064 (4)	0.007 (5)

### (3aS,4R,5S,7aR)-7-(But-3-en-1-yn-1-yl)-2,2-dimethyl-3a,4,5,7a-tetrahydro-2H-1,3-benzodioxole-4,5-diol Geometric parameters (Å, º)

**Table:**

C2—O2	1.437 (7)	C5—C7	1.457 (8)
C2—C1	1.492 (6)	O2—H2A	0.8200
C2—C3	1.508 (7)	C6—H6	0.9300
C2—H2	0.9800	C7—C8	1.168 (9)
C1—O1	1.414 (7)	C8—C9	1.433 (12)
C1—C6	1.502 (8)	C9—C10	1.264 (16)
C1—H1A	0.9800	C9—H9	0.9300
O1—H1	0.8200	C10—H10A	0.9300
O3—C3	1.420 (5)	C10—H10B	0.9300
O3—C11	1.422 (7)	C11—C12	1.502 (9)
O4—C11	1.431 (6)	C11—C13	1.521 (10)
O4—C4	1.436 (6)	C12—H12A	0.9600
C3—C4	1.538 (7)	C12—H12B	0.9600
C3—H3	0.9800	C12—H12C	0.9600
C4—C5	1.520 (7)	C13—H13A	0.9600
C4—H4	0.9800	C13—H13B	0.9600
C5—C6	1.324 (8)	C13—H13C	0.9600
			
O2—C2—C1	108.9 (4)	C2—O2—H2A	109.5
O2—C2—C3	107.6 (5)	C5—C6—C1	123.4 (5)
C1—C2—C3	109.1 (4)	C5—C6—H6	118.3
O2—C2—H2	110.4	C1—C6—H6	118.3
C1—C2—H2	110.4	C8—C7—C5	177.7 (7)
C3—C2—H2	110.4	C7—C8—C9	176.9 (10)
O1—C1—C2	111.0 (4)	C10—C9—C8	128.6 (13)
O1—C1—C6	107.3 (5)	C10—C9—H9	115.7
C2—C1—C6	110.1 (4)	C8—C9—H9	115.7
O1—C1—H1A	109.4	C9—C10—H10A	120.0
C2—C1—H1A	109.4	C9—C10—H10B	120.0
C6—C1—H1A	109.4	H10A—C10—H10B	120.0
C1—O1—H1	109.5	O3—C11—O4	106.5 (4)
C3—O3—C11	109.3 (4)	O3—C11—C12	109.1 (5)
C11—O4—C4	103.6 (4)	O4—C11—C12	108.5 (5)
O3—C3—C2	109.5 (4)	O3—C11—C13	107.7 (6)
O3—C3—C4	103.5 (4)	O4—C11—C13	112.4 (5)
C2—C3—C4	113.2 (4)	C12—C11—C13	112.3 (6)
O3—C3—H3	110.1	C11—C12—H12A	109.5
C2—C3—H3	110.1	C11—C12—H12B	109.5
C4—C3—H3	110.1	H12A—C12—H12B	109.5
O4—C4—C5	108.0 (4)	C11—C12—H12C	109.5
O4—C4—C3	104.9 (4)	H12A—C12—H12C	109.5
C5—C4—C3	115.8 (4)	H12B—C12—H12C	109.5
O4—C4—H4	109.3	C11—C13—H13A	109.5
C5—C4—H4	109.3	C11—C13—H13B	109.5
C3—C4—H4	109.3	H13A—C13—H13B	109.5
C6—C5—C7	122.4 (5)	C11—C13—H13C	109.5
C6—C5—C4	119.6 (5)	H13A—C13—H13C	109.5
C7—C5—C4	117.7 (5)	H13B—C13—H13C	109.5
			
O2—C2—C1—O1	65.4 (6)	C2—C3—C4—C5	−19.9 (6)
C3—C2—C1—O1	−177.4 (5)	O4—C4—C5—C6	−127.6 (5)
O2—C2—C1—C6	−175.9 (5)	C3—C4—C5—C6	−10.4 (7)
C3—C2—C1—C6	−58.7 (6)	O4—C4—C5—C7	58.1 (6)
C11—O3—C3—C2	−122.7 (5)	C3—C4—C5—C7	175.4 (5)
C11—O3—C3—C4	−1.7 (5)	C7—C5—C6—C1	179.1 (5)
O2—C2—C3—O3	−72.8 (5)	C4—C5—C6—C1	5.1 (9)
C1—C2—C3—O3	169.2 (4)	O1—C1—C6—C5	151.3 (6)
O2—C2—C3—C4	172.3 (4)	C2—C1—C6—C5	30.3 (8)
C1—C2—C3—C4	54.2 (6)	C3—O3—C11—O4	22.5 (6)
C11—O4—C4—C5	156.9 (4)	C3—O3—C11—C12	139.5 (5)
C11—O4—C4—C3	32.8 (5)	C3—O3—C11—C13	−98.3 (5)
O3—C3—C4—O4	−19.4 (5)	C4—O4—C11—O3	−34.5 (5)
C2—C3—C4—O4	99.0 (5)	C4—O4—C11—C12	−151.9 (6)
O3—C3—C4—C5	−138.4 (4)	C4—O4—C11—C13	83.2 (6)

### (3aS,4R,5S,7aR)-7-(But-3-en-1-yn-1-yl)-2,2-dimethyl-3a,4,5,7a-tetrahydro-2H-1,3-benzodioxole-4,5-diol Hydrogen-bond geometry (Å, º)

**Table:**

*D*—H···*A*	*D*—H	H···*A*	*D*···*A*	*D*—H···*A*
O1—H1···O1^i^	0.82	2.00	2.803 (3)	167
O2—H2*A*···O3^ii^	0.82	2.19	2.995 (6)	168

Symmetry codes: (i) −*x*+1/2, *y*−1/2, −*z*+1; (ii) −*x*+1, *y*, −*z*+1.

## References

[bb1] Bourhis, L. J., Dolomanov, O. V., Gildea, R. J., Howard, J. A. K. & Puschmann, H. (2015). *Acta Cryst.* A**71**, 59–75. 10.1107/S2053273314022207 PMC4283469 25537389

[bb20] Bruker (2018). *APEX3* and *SAINT*. Bruker AXS Inc., Madison, Wisonsin, USA.

[bb2] Buckler, J. N., Taher, E. S., Fraser, N. J., Willis, A. C., Carr, P. D., Jackson, C. J. & Banwell, M. G. (2017). *J. Org. Chem.* **82**, 15, 7869–7886. 10.1021/acs.joc.7b01062 28671462

[bb3] Cremer, D. & Pople, J. A. (1975). *J. Am. Chem. Soc.* **97**, 1354–1358.

[bb4] Dolomanov, O. V., Bourhis, L. J., Gildea, R. J., Howard, J. A. K. & Puschmann, H. (2009). *J. Appl. Cryst.* **42**, 339–341.

[bb5] Groom, C. R., Bruno, I. J., Lightfoot, M. P. & Ward, S. C. (2016). *Acta Cryst.* B**72**, 171–179. 10.1107/S2052520616003954 PMC4822653 27048719

[bb6] Hookins, D. R., Burns, A. R. & Taylor, R. J. K. (2011). *Eur. J. Org. Chem.* pp. 451–454.

[bb21] Hübschle, C. B., Sheldrick, G. M. & Dittrich, B. (2011). *J. Appl. Cryst.* **44**, 1281–1284. 10.1107/S0021889811043202 PMC3246833 22477785

[bb7] Jiang, M.-Y., Li, Y., Wang, F. & Liu, J.-K. (2011). *Phytochemistry*, **72**, 923–928. 10.1016/j.phytochem.2011.03.011 21477823

[bb8] Jiang, M.-Y., Zhang, L., Liu, R., Dong, Z.-J. & Liu, J.-K. (2009). *J. Nat. Prod.* **72**, 1405–1409. 10.1021/np900182m 19624146

[bb9] Kim, H.-J., Vinale, F., Ghisalberti, E. L., Worth, C. M., Sivasithamparam, K., Skelton, B. W. & White, A. H. (2006). *Phytochemistry*, **67**, 2277–2280. 10.1016/j.phytochem.2006.07.022 16942784

[bb10] Krause, L., Herbst-Irmer, R., Sheldrick, G. M. & Stalke, D. (2015). *J. Appl. Cryst.* **48**, 3–10. 10.1107/S1600576714022985 PMC4453166 26089746

[bb11] Macías, M. A., Suescun, L., Pandolfi, E., Schapiro, V., Tibhe, G. D. & Mombrú, Á. W. (2015). *Acta Cryst.* E**71**, 1013–1016. 10.1107/S2056989015014590 PMC4555417 26396837

[bb12] Nagata, T., Ando, Y. & Hirota, A. (1992). *Biosci. Biotechnol. Biochem.* **56**, 810–811. 10.1271/bbb.56.810 27286214

[bb13] Pandolfi, E., Schapiro, V., Heguaburu, V. & Labora, M. (2013). *Curr. Org. Synth.* **10**, 2–42.

[bb14] Parsons, S., Flack, H. D. & Wagner, T. (2013). *Acta Cryst.* B**69**, 249–259. 10.1107/S2052519213010014 PMC3661305 23719469

[bb15] Peixoto de Abreu Lima, A., Suescun, L., Pandolfi, E. & Schapiro, V. (2019). *New J. Chem.* **43**, 3653–3655. 10.1107/S2056989024009733 PMC11660465 39712156

[bb16] Sheldrick, G. M. (2015). *Acta Cryst.* C**71**, 3–8.

[bb17] Taher, E. S., Guest, P., Benton, A., Ma, X., Banwell, M. G., Willis, A. C., Seiser, T., Newton, T. W. & Hutzler, J. (2017). *J. Org. Chem.* **82**, 1, 211–233. 10.1021/acs.joc.6b02372 28026176

[bb18] Tibhe, G. D., Macías, M. A., Schapiro, V., Suescun, L. & Pandolfi, E. (2018). *Molecules*, **23**, 1653. 10.3390/molecules23071653 PMC6100410 29986401

[bb19] Vila, M. A., Brovetto, M., Gamenara, D., Bracco, P., Zinola, G., Seoane, G., Rodríguez, S. & Carrera, I. (2013). *J. Mol. Catal. B Enzym.* **96**, 14–20.

